# Estrogenic Plant Extracts Reverse Weight Gain and Fat Accumulation without Causing Mammary Gland or Uterine Proliferation

**DOI:** 10.1371/journal.pone.0028333

**Published:** 2011-12-07

**Authors:** Elise F. Saunier, Omar I. Vivar, Andrea Rubenstein, Xiaoyue Zhao, Moshe Olshansky, Scott Baggett, Richard E. Staub, Mary Tagliaferri, Isaac Cohen, Terence P. Speed, John D. Baxter, Dale C. Leitman

**Affiliations:** 1 Bionovo Inc., Emeryville, California, United States of America; 2 Department of Nutritional Science and Toxicology, University of California, Berkeley, California, United States of America; 3 Division of Bioinformatics, The Walter and Eliza Hall Institute of Medical Research, Parkville, Victoria, Australia; 4 Department of Statistics, University of California, Berkeley, California, United States of America; 5 Diabetes Center and Cancer Research Unit, The Methodist Hospital Research Institute, Houston, Texas, United States of America; Ecole Normale Supérieure de Lyon, France

## Abstract

Long-term estrogen deficiency increases the risk of obesity, diabetes and metabolic syndrome in postmenopausal women. Menopausal hormone therapy containing estrogens might prevent these conditions, but its prolonged use increases the risk of breast cancer, as wells as endometrial cancer if used without progestins. Animal studies indicate that beneficial effects of estrogens in adipose tissue and adverse effects on mammary gland and uterus are mediated by estrogen receptor alpha (ERα). One strategy to improve the safety of estrogens to prevent/treat obesity, diabetes and metabolic syndrome is to develop estrogens that act as agonists in adipose tissue, but not in mammary gland and uterus. We considered plant extracts, which have been the source of many pharmaceuticals, as a source of tissue selective estrogens. Extracts from two plants, *Glycyrrhiza uralensis* (RG) and *Pueraria montana* var. *lobata* (RP) bound to ERα, activated ERα responsive reporters, and reversed weight gain and fat accumulation comparable to estradiol in ovariectomized obese mice maintained on a high fat diet. Unlike estradiol, RG and RP did not induce proliferative effects on mammary gland and uterus. Gene expression profiling demonstrated that RG and RP induced estradiol-like regulation of genes in abdominal fat, but not in mammary gland and uterus. The compounds in extracts from RG and RP might constitute a new class of tissue selective estrogens to reverse weight gain, fat accumulation and metabolic syndrome in postmenopausal women.

## Introduction

Menopause is associated with a profound drop in levels of estrogens in women. This leads to estrogen deficiency which initiates early symptoms including hot flashes, mood swings and vaginal dryness, and contributes to long-term conditions such as osteoporosis, and possibly other chronic conditions, including cardiovascular disease, obesity, type 2 diabetes, metabolic syndrome and Alzheimer's disease. Postmenopausal women are frequently treated with menopausal hormone therapy (MHT) containing estrogens that are very effective at preventing hot flashes, urogenital atrophy and osteoporosis [1]. The Women's Health Initiative (WHI) trial [2], [3], Heart and Estrogen/Progestin Replacement Study [4] and Nurses' Health Study [5] also found that MHT decreases the risk for type 2 diabetes. Other studies indicate that MHT decreases weight gain [6], [7] and prevents redistribution of fat to the abdominal cavity [8], [9]. Despite these important benefits the WHI demonstrated that MHT increases the risk of breast cancer, blood clots and cardiovascular disease [1], [10]. While controversial, the potential l adverse effects of estrogens have led to a large decline in MHT use [11] and an intense pursuit to develop safer estrogens in MHT for long-term therapy.

Estrogens in MHT were introduced many years prior to the elucidation of factors involved in estrogen receptor signaling. Major discoveries include the identification of two estrogen receptor subtypes, multiple classes of DNA regulatory elements that estrogen receptors bind to in genes, and numerous coregulator proteins and transcription factors that interact with ERs to alter chromatin structure to regulate gene transcription [12]. These exciting discoveries might make it possible to develop estrogen mimetics that are more selective and do not require the addition of progestins, which exacerbate adverse effects of estrogens on breast cells [13].

The major problem with current estrogens used in MHT is that they are agonists in all tissues and are therefore non-selective [14]. A variety of ER subtype selective estrogens have been identified. 4,4′,4″-(4-Propyl-[1*H*]-pyrazole-1,3,5-triyl)*tris*phenol (PPT) is a highly selective ERα agonist [15]. Multiple synthetic ERβ-selective agonists have also been developed [16] with diarylpropionitrile (DPN) and ERB-041 being the most studied [17], [18]. ERβ-selective agonists have been discovered from plants, including MF101 and liquiritigenin [19], [20]. ERβ-selective compounds are a very attractive alternative to non-selective estrogens currently in MHT because of the anti-proliferative and anti-tumor properties of ERβ [21]–[24], and the finding that MF101 produced a significant reduction in hot flashes and night sweats [25]. However, there is significant data demonstrating that ERα mediates the beneficial metabolic effects of estrogens in adipose tissues [26], [27]. Estradiol (E2) has powerful antidiabetic (with improved insulin sensitivity and glucose tolerance) and weight-lowering effects in ovariectomized mice and rats, and in several rodent models of spontaneous type 2 diabetes [28], [29]. ERα knockout (ERKO) mice have increased weight gain, greater adipose tissue, insulin resistance and impaired glucose tolerance [30], [31]. The selective ERα agonist, PPT reduced weight gain and fat accumulation in normal mice [32], [33] and improved glucose tolerance and insulin sensitivity in obese mice [34]. In contrast, no increase in weight is observed in ERβ knockout (BERKO) mice and the ERβ agonist, DPN does not alter total body weight gain in rats [35]. Although it is conceivable that the role of ERα and ERβ might differ in humans, the rodent studies indicate that the beneficial effects of estrogens on fat metabolism are likely mediated by ERα, rather than ERβ.

One strategy to exploit the beneficial metabolic effects of ERα is to identify tissue selective ERα drugs that act as agonists in adipose tissue, but not in the mammary gland (MG) or uterus. Massive efforts have been directed at identifying estrogens with greater selectivity. These have employed large scale high throughput screenings, X-ray crystallographic approaches and other methods. Besides non-selective estrogens the only other class of estrogenic drugs available for menopausal women are the selective estrogen receptor modulators (SERMs), such as raloxifene that possess mixed agonist/antagonist activity [36]. Many plants contain phytoestrogens which, like estradiol, transactivate estrogen receptors. Some phytoestrogens exhibit selectivity for ER subtypes [37]. We therefore searched for compounds in plants that have yielded many pharmaceuticals and are popular among women [38] to discover estrogens that might exhibit tissue specific effects which could prevent metabolic complications during menopause. Here we report the discovery of plant extracts that have estrogenic activity and reverse weight gain and fat accumulation without eliciting untoward effects on mammary gland or uterus in mice.

## Materials and Methods

### Plant extracts

#### Preparation of RG

Five kilograms of ground, dried roots of *Glycyrrhiza uralensis* Fischer or *Glycyrrhiza glabra* L.; (Fabaceae) was extracted twice with 25 liters of 8∶2 ethanol—water for 8 h and overnight at room temperature with constant stirring. The extract was suction filtered through Whatman #4 filter paper, and the combined filtrates were concentrated under reduced pressure at 50°C to approximately 2 liters to remove the ethanol. The aqueous fraction was freeze dried to produce a stable powder that could be diluted to a specified concentration for testing.

#### Preparation of RP

Five kilograms of ground, dried roots of *Pueraria montana* (Lour.) Merr. var. *lobata* (Willd.) Maesen & S. Almeida (Fabaceae) was extracted twice with 25 liters of 8∶2 ethanol—water for 8 h and overnight at room temperature with constant stirring. The extract was suction filtered through Whatman #4 filter paper, and the combined filtrates were concentrated under reduced pressure at 50°C to approximately 2 liters to remove the ethanol. Water was added to increase the volume to 4 liters, and the aqueous fraction was partitioned four times with an equal volume (4 L) of ethyl acetate. The ethyl acetate layers were combined and concentrated to dryness under reduced pressure. The remaining solids were resuspended from the round bottom flask with a combination of water and ethanol, and the solution was freeze dried to produce a stable powder that could be diluted to a specified concentration for testing.

### Cell culture, transfection, and luciferase assays

Human U2OS osteosarcoma, MCF-7 breast cancer and ECC-1 endometrial cancer cell lines were obtained from the American Type Culture Collection (Manassas, VA). U2OS cells expressing a doxycycline-inducible ERα cDNA were previously described [39]. All cell lines were maintained and subcultured as previously described [40]. Transfections were carried out with a Bio-Rad gene pulser as previously described [40]. Briefly, U2OS cells were electroporated with 3 µg of ERE and NKG2E tk-Luciferase (tk-Luc) reporter vector and 1 µg of an expression vector for ERα. ECC-1 cells were electroporated with 3 µg of ERE tk-Luc. After electroporation, the cells were plated and treated with E2, RG, or RP for 18 h. Cells were then solubilized and luciferase activity was determined with an assay kit (Promega, Madison, WI). Experiments were performed at least three times and the mean ± S.E.M. was calculated.

### ERα binding assay

Binding to ERα was determined using fluorescence polarization-based ER competitor assays per the manufacturer's protocol (Invitrogen, Carlsbad, CA). The 2X Fluormone™ ES2/ERα complex was added to a dilution series of RG and RP to generate competition curves. The polarization was plotted against the concentration of RG and RP. The concentration of RG and RP that results in a half maximum shift in polarization equals the IC50.

### Xenograft studies in nude mice

MCF-7 cells were grown in DMEM/F-12 medium containing 4% stripped fetal bovine serum and re-suspended in sterile PBS∶Matrigel (1∶1). MCF7 cells (250,000 cells) were injected into the mammary fat pad (MG #4; both sides) of 8 week old CD-1 nude mice (Charles River, Wilmington, MA). The mice were randomly separated in ten groups. Control mice were treated with vehicle. E2 treated mice received a subcutaneous mini-pump (Alzet, Cupertino, CA) containing 0.25 mg E2. Eight groups were treated orally, daily, five times a week for 4 weeks with 40, 80, 160 or 320 mg/day RG or RP separately. The tumor size was measured after 4 weeks. All animal protocols were approved by the Institutional Animal Care and Use Committee of Bionovo. All animal experiments were carried out in strict accordance with the recommendations in the Guide for the Care and Use of Laboratory Animals of the National Institutes of Health.

### Weight studies in mice

Twenty 10 week old, ovariectomized C57Bl/6 female mice (Jackson Laboratory, Bar Harbor, ME) were fed with a high fat diet (Research Diets, Inc., New Brunswick, NJ) ad libidum for 7 weeks to increase body weight. After gaining weight, the mice were randomly separated in four groups of five mice. Two groups were treated orally, five times a week for 6 weeks with 80 mg RG or RP while being maintained on a HFD. Control mice were treated with a continuous infusion using osmotic Alzet pumps containing vehicle. E2 treated mice received pumps containing 0.25 mg E2 in vehicle. Body weight was recorded weekly. At the end of the experiment, #4 mammary glands were collected and weighed. One gland of each pair was prepared for whole mounts and the other one for histology and RNA extraction. The abdominal fat depots were also collected, weighed and extracted for RNA. Finally, the uterus was collected and weighed, and used for RNA extraction. The experiment was reproduced and gave similar results. The data presented are representative of both experiments.

### Mammary gland whole mounts

Mammary gland whole mounts were prepared as described previously [41]. Briefly, #4 mammary glands were dissected away from subcutaneous tissues and fixed in freshly prepared Methacorn solution (60% methanol, 30% chloroform, and 10% acetic acid) for 48 h, dehydrated, skimmed with acetone for 48 h, rehydrated, and stained with iron hematoxylin for 2 h before washing and Histo-Clear conservation (National Diagnostics, Somerville, NJ) as described [41]. The mammary gland whole mounts were examined by light microscopy to evaluate the mammary structures.

### Microarrays

Abdominal fat, #4 mammary glands and uterus tissues from the mice described above were collected in RNAlater RNA Stabilization Reagent (Qiagen). Total RNA was isolated with the Aurum total RNA fatty and fibrous tissue kit (Bio-Rad, Hercules, CA) per the manufacturer's protocol. RNA was first quantified by standard spectrophotometry, and then qualitatively evaluated by capillary electrophoresis employing the Experion system (Bio-Rad, Hercules, CA). Biotin-labeled cRNA samples were prepared with 400 ng of total RNA template. Following synthesis and purification, the biotin-labeled samples were evaluated by both 260/280 absorbance spectrophotometry and capillary electrophoresis. The final labeled cRNA samples were hybridized overnight against MouseWG-6 BeadChip arrays containing more than 45,200 probes (Illumina) by the University of California, San Francisco Genomics Core.

The raw intensities data was converted into text files by Illumina BeadStudio software package and then analyzed using the BioConductor limma package. In particular, the data was background corrected by Normal+Exponential model using negative controls and then quantile normalized. A linear model was fit by limma and p-values were computed based on moderated t-test and adjusted for multiple testing by applying Benjamini-Hochberg correction. Genes with adjusted p-values<0.05 were considered statistically significant. All microarray data will be deposited in a publicly available database.

### Real-time RT-PCR

Total RNA extracted from tissues was reverse transcribed using the iScript cDNA Synthesis Kit (Bio-Rad, Hercules, CA). Real-time quantitative PCR was performed using SYBR Green Supermix with an iCycler thermal cycler (Bio-Rad) as previously described [42]. Experiments were performed at least three times. The following PCR primers were used:

MUP1 Forward 5′-CCCAGAGAGTATATAAGGACAAGCAAAGG-3′; Reverse 5′-AGTATGCCATTCCCCATTAATCTTTTCTAC-3′; GPX3 Forward 5′-GTGAGCGCGATGGCCGTGTA-3′; Reverse 5′-ACCACAGACGGGCTCAGGGG-3′; LCN2 Forward 5′-TGGGGTCCTGCAGAGCCAGG-3′; Reverse 5′-CCTGCCCCGGAACTGATCGC-3′; CD8B Forward 5′-ACTTCTGCGCGACGGTTGGG-3′; Reverse 5′-GGGTGGGGGAACGGGCATTG-3′; CD79B Forward 5′-GGAGACAAGCTGCAGCCCAGG-3′; Reverse 5′-CAACAGCCAGTGGCAGGGCA-3′; LEP Forward 5′-GAGCACAAGGAGGGGCCAGC-3′; Reverse 5′-CGCAGCGAAGCGTGTACCCA-3′.

## Results

### Plant extracts activate transcription through ERα

Eighteen plant extracts (PEs) used historically for treating menopausal-related conditions [43] were screened for ERα activity by transfecting U2OS cells with the classic estrogen responsive element (ERE) upstream of the minimal thymidine kinase promoter linked to the luciferase reporter gene (ERE tk-Luc) and an expression vector for human ERα. Based on these screens we selected PEs from Radix *Glycyrrhiza uralensis* (RG) and Radix *Pueraria montana* var. *lobata* (RP) for further studies because they exhibited ERα-mediated activation of ERE tk-Luc.

**Figure 1:**
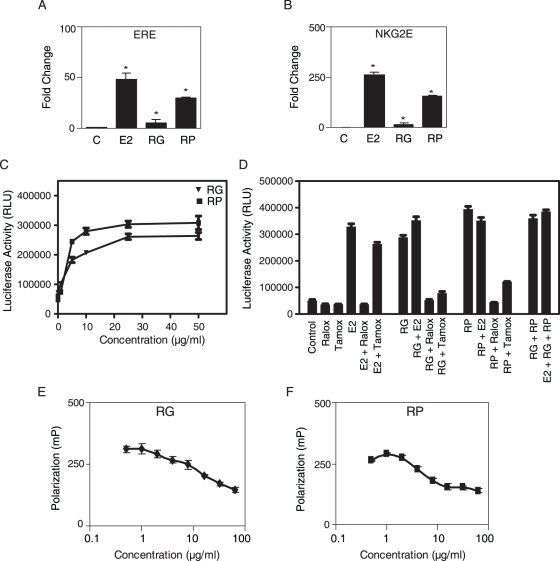
RG and RP activate transcription through ERα. Human U2OS osteosarcoma cells were transfected with (A) ERE tk-Luc or (B) NKG2E tk-Luc reporter vectors and an expression vector for ERα. After transfection, the cells were treated for 18 h with 10 nM E2, 10 µg/ml RG or 10 µg/ml RP and luciferase activity was measured. (C) ECC-1 cells were transfected with ERE tk-Luc and treated with increasing amounts of RG and RP for 18 hours and then luciferase activity was measured. (D) ECC-1 cells were transfected with ERE tk-Luc and treated with 1 nM E2, 1 µM raloxifene, 1 µM tamoxifen, 25 µg/ml RG or 25 µg/ml RP individually or in combination for 18 hours and then luciferase activity was measured. Each data point is the average of triplicate samples ± S.E.M. (E) RG and (F) RP binding to purified ERα was assayed by fluorescence polarization-based ER competitor assays. Serial dilutions of RG and RP were tested and the IC50 determined. * represents a significant difference from control (P<0.05, t-test).

In addition to the classic ERE, ERα activity of RG and RP was examined using a more complex ERα responsive regulatory element derived from the NKG2E promoter, which requires a collaboration between c-jun, heat-shock factor 2, and CCAAT/enhancer-binding protein beta and a variant ERE for full activation by E2 [44]. RG and RP were less active than E2 in U2OS osteosarcoma cells transfected with an expression vector for ERα [39], but they produced a significant activation of ERE (Fig. 1*A*) and NKG2E (Fig. 1*B*) tk-Luc reporters. These data demonstrate that RG and RP have ERα activity with different types of estrogen response elements.

### RG and RP act differently than SERMs

To investigate if RG and RP behave similarly to mixed agonist/antagonist estrogens like the SERMs, raloxifene and tamoxifen, we examined their effects on ERE tk-Luc in the absence and presence of E2 in human ECC-1 endometrial cancer cells that express endogenous ERα [45]. RG and RP produced a dose-dependent activation of ERE tk-Luc. The activation was first observed at 1 µg/ml, and a 5–6 fold maximal activation occurred at 25 µg/ml (Figure 1*C*). Raloxifene and tamoxifen did not activate ERE tk-Luc in ECC-1 cells (Figure 1*D*), but raloxifene antagonized the activation by E2, RG and RP and tamoxifen antagonized the activation by RG and RP. Unlike the SERMs, RG and RP did not antagonize E2 activation of ERE tk-Luc (Figure 1*D*). At higher doses, RG, RP and E2 produced an equivalent activation of the ERE in ECC-1 cells. Thus, RG and RP act exclusively as agonists, whereas the raloxifene and tamoxifen act as an antagonist on the ERE in these cells.

### RG and RP bind to ERα

We tested RG and RP binding to ERα by fluorescence polarization-based ER competitor assays. Both RG and RP effectively competed with fluorescent E2 for binding to purified full-length ERα [EC50s were 16 µg/ml and 5.5 µg/ml for RG and RP, respectively (Figure 1*E and F*)]. These results demonstrate that compounds in RG and RP bind to ERα.

### ERα is required for RG and RP regulation of genes in U2OS cells

Effects of RG and RP on endogenous genes were examined in human U2OS cells stably transfected with doxycycline-inducible expression for ERα. We previously demonstrated that E2 regulated numerous genes in these cells [46]. In the absence of doxycycline ERα is not expressed [39] and only 5 genes were regulated by RG and 33 by RP (Table 1). In contrast, after induction with doxycycline RG regulated 347 genes and RP regulated 1,598 genes in U2OS-ERα cells (Supplemental Table 1). The reporter assays, competitive binding study and gene expression data indicate that ERα mediates the effects of RG and RP.

**Table 1:** RG and RP regulate many genes only when U2OS cells express ERα.

Treatment	Number of genes regulated
−dox RG vs. −dox Control	5
−dox RP vs. −dox Control	33
+dox RG vs. +dox Control	347
+dox RP vs. +dox Control	1598

U2OS cells stably transfected with a doxycycline-inducible ERα were maintained for 24 h in the absence of doxycycline (−dox) or presence of 1 µg/ml doxycycline (+dox). The cells were then treated without (Control) or with RG or RP for 18 h. The number of genes regulated was defined by a statistically significant (p<0.05) 2-fold or greater activation or a repression greater than 50%. The microarrays were done in triplicate.

### RG and RP reverse weight gain in high fat diet fed mice

**Figure 2:**
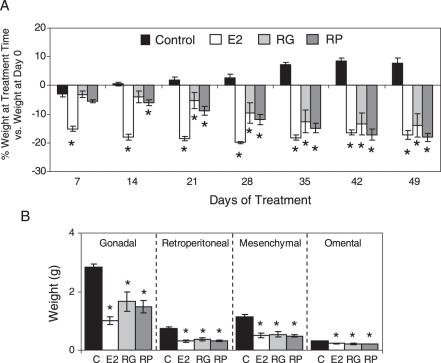
RG and RP reverse weight gain and fat accumulation in mice fed a high fat diet. Ten week old ovariectomized C57Bl/6 female mice were placed on a HFD for 50 days to increase body weight and abdominal fat. After 50 days on a HFD the mice gained about 40% in body weight and then they were treated orally for 7 weeks with 80 mg/day RG or RP while being maintained on a HFD. E2 treated mice received a subcutaneous pump containing E2. Each mouse was weighed weekly. (A) Percentage of weight change at the duration of treatment to the initial body weight at day 0 when the drugs were started (50 days on HFD). Each data are expressed as average ± S.E.M. (P<0.05, t-test). * represents significant differences after treatment to day 0 weight (P<0.05, t-test). (B) The weights of the gonadal, retroperitoneal, mesenchymal and omental fat depots were measured for each mouse. The data represent the average weight per treatment in each mouse (n = 5±S.E.M.). * represents significant differences in treated mice compared to control mice (P<0.05, t-test).

We examined if RG and RP reverses weight gain and fat accumulation using a high fat diet (HFD) fed mouse model. Ten week old ovariectomized C57Bl/6 female mice were fed a HFD for 50 days to increase body weight and abdominal fat. Once the mice gained weight they were randomly divided into four groups that received: (Group 1) only a HFD (control mice); (Group 2) both E2 and HFD (positive control); (Group 3) HFD and RG orally for 49 days; and (Group 4) HFD and RP orally, 49 days. The total length of the study was 109 days. From day 50 to 109 the control mice continued to gain additional 8% of body weight (Fig. 2*A*). In contrast, E2 treated mice started to lose weight significantly after one week, and continued to lose weight for the duration of the experiment (−17% at day 49 of treatment; P<0.05, t-test). A significant weight loss was first observed after 14 days treatment with RP and 21 days with RG. Both groups continued to lose weight during the 49 days of treatment (P<0.05, t-test). At the end of study the mice treated with RG and RP lost 14% and 18%, respectively, from their pre-treatment body weight at day 0, which was similar to mice treated with E2 (Fig. 2*A*). RG and RP also produced a significant reduction in the weight of gonadal, retroperitoneal, mesenchymal and omental fat depots with a similar magnitude to E2 (Fig. 2*B*). RG and RP mice had a feeding behavior similar to the control mice (Supplemental Table 2), demonstrating that the weight loss was not due to a decrease in food intake.

### RG and RP do not mimic E2 on mammary gland and uterus

**Figure 3:**
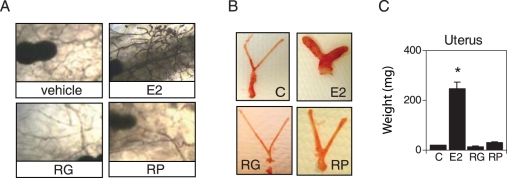
RG and RP do not mimic estradiol in mammary gland and uterus. The mammary glands and uterus were collected from the mice used to study body weight and fat weight (Figure 2) at the end of the 7 weeks of treatment. Eight week old mature mice were ovariectomized. At 10 weeks of age, mice were fed a high fat diet for 7 weeks and then treatment with E2, RG and RP was started. The high fat diet was continued during treatment. After 7 weeks of treatment the same animals were used for body weight, fat weight, mammary gland and uterus study. (A) Representative mammary gland whole mounts are shown. (B) Representative pictures of the uterus are shown. (C) The weights of uterus were measured for each mouse. The data represent the average weight per treatment (n = 5 mice±S.E.M), normalized to the average weight of control mice * represents significant differences compared to estradiol treated mice (P<0.05, t-test).

Potential proliferative effects of RG and RP on the MG were assessed by examining their morphology in whole mounts after 49 days of treatment. E2 increased the number of branching ductal structures compared to control mice (Figs. 3*A*). The MGs of RG- and RP-treated mice had a similar phenotype to control mice with few ductal structures indicating that they did not stimulate epithelial cell proliferation and differentiation (Fig. 3*A*). Uterine size (Fig. 3*B*) and weights (Fig. 3*C*) in the RG- and RP-treated groups were similar to the control mice, whereas E2 produced a marked increase in uterine size and weight. These data demonstrate that unlike E2, RG and RP exert favorable tissue-specific effects that reverse body weight and fat accumulation without causing proliferative effects on the MG and uterus.

### Unlike E2, RG and RP gene regulation is adipose tissue specific

To examine if RG and RP produce tissue specific effects at the genomic level, we compared gene expression profiles in gonadal fat, MG and uterus in response to E2, RG and RP. In gonadal fat, E2, RG and RP each regulated over 1,000 genes (Table 2). The genes regulated are listed in Supplemental Table 2. 24% of genes regulated by RG and RP overlapped with genes regulated with E2 indicating that RG and RP can regulate the activity of ER in adipose tissue. In contrast, RG and RP regulated 0 or 22 genes, respectively in the MG compared to 479 genes regulated by E2. The lack of effects on gene regulation by RG and RP is consistent with the absence of proliferation in the MG observed in whole mounts after treatment. In the uterus E2 regulated 2239 genes, whereas RG regulated 229 genes and RP regulated 664 genes (Supplemental Table 3). The overall profile of gene regulation clearly demonstrates the tissue specificity of RG and RP.

**Table 2:** RG and RP gene regulation profile mimics estradiol in fat only.

Tissue	Treatment	Induced genes	Repressed genes	Total genes regulated	Common E2	% of E2
Gonadal Fat	E2	662	517	1179		
Gonadal Fat	RG	270	768	1038	287	24.34
Gonadal Fat	RP	332	724	1046	284	24.09
Mammary Gland	E2	208	271	479		
Mammary Gland	RG	0	0	0	0	0
Mammary Gland	RP	18	4	22	10	2.09
Uterus	E2	1250	989	2239		
Uterus	RG	6	223	229	92	4.11
Uterus	RP	259	405	664	248	11.08

Gene expression from gonadal fat, mammary gland and uterus was analyzed by microarray. The number of genes induced and repressed by estradiol (E2), RG and RP and the total number of gene regulated per tissue are specified compared to control mice. The number of genes regulated by RG and RP that are commonly regulated by E2 are shown (common E2) and specified as a percentage of E2 regulated genes (% of E2).

**Figure 4:**
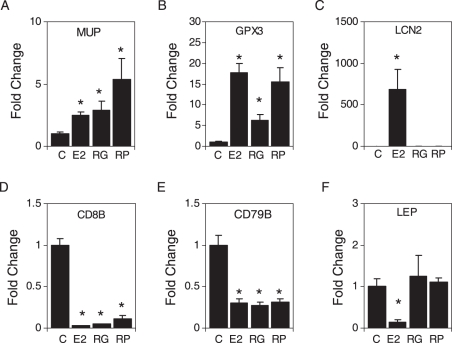
RG and RP effects on expression of genes selected from microarrays. Relative mRNA levels for (A) MUP, (B) GPX3, (C) LCN2, (D) CD8B, (E) CD79B and (F) LEP in gonadal fat tissue from control (C), E2, RG and RP treated mice as determined by real-time PCR normalized to β-actin. Each data point is the average of four samples ± S.E.M. * represents a significant difference from control (P<0.05).

Some genes regulated by E2, RG and RP in adipose tissue have been implicated in obesity and were verified by real time PCR. The major urinary protein (MUP) (Fig. 4*A*) and glutathione peroxidase 3 (GPX3) (Fig. 4*A*) genes were up-regulated by all three treatments. The lipocalin 2 (LCN2) gene was up-regulated by E2, but not by RG and RP (Fig. 4*C*). Expression of CD8B (Fig. 4*D*) and CD79B (Fig. 4*E*) antigens were down-regulated by all three treatments. The leptin (LEP) gene was down-regulated by E2, but not by RG and RP (Fig. 4*F*).

### RG and RP do not promote estrogen-dependent MCF-7 breast cancer tumor growth

**Figure 5:**
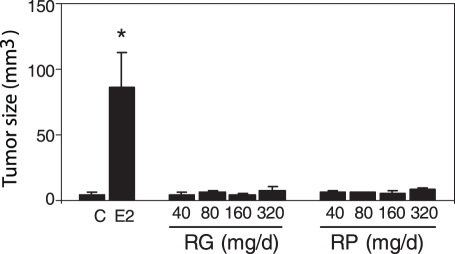
RG and RP do not stimulate tumor growth of MCF-7 cells in xenografts. Eight week old nude mice were injected with MCF-7 cells in the mammary fat pad of both MG #4. The mice were treated with vehicle control (C), 0.25 mg E2 per pump or RG and RP at 40, 80, 160 and 320 mg daily. The data represent the average tumor size per treatment ± S.E.M. for measurable tumors. * represents significant differences in treated mice compared to control mice (P<0.05, t-test).

We analyzed potential estrogenic effects of RG and RP in a human cell system using the estrogen dependent MCF-7 breast cancer xenograft model. Eight week old ovariectomized nude female mice were implanted with MCF-7 cells in the mammary fat pad. The mice were randomly divided into ten groups. The first group received vehicle (control mice) and the second group received E2 using a mini-osmotic pump. The other groups were treated orally for 4 weeks with 40, 80, 160 or 320 mg/day of RG or RP separately. The E2 treated mice developed tumor nodules in every MG pad that were always large enough to be measured in three dimensions (Fig. 5; P<0.05, t-test). In mice treated with of RG and RP the tumors typically did not develop, but when they did they were minimal in size and not different from controls at all doses tested. Thus, RG and RP do not have estrogenic activity in human MCF-7 cell xenograft model.

## Discussion

The current studies illustrate the issue with E2 acting in a non-tissue selective manner leading to both beneficial and adverse effects as observed in clinical trials with MHT [1]. Our study demonstrates the non-tissue selective effect of E2 by showing that it had agonist activity in mouse adipose tissue, MG and uterus. To overcome this non-selective agonist action, we identified RG and RP plant extracts, and demonstrated that they exhibit tissue selective estrogenic activity. RG and RP had the same favorable agonist activity as E2 on metabolic parameters, which was demonstrated by a reversal of weight gain and fat accumulation. These beneficial effects were previously shown to be mediated by ERα in mice [30]. However, RG and RP did not promote other known actions mediated by ERα, such as the proliferation of mouse MG cells, increasing uterine growth or stimulating MCF-7 breast cancer tumor formation in a mouse xenograft model. These findings demonstrate that RG and RP exhibit estrogenic activity in adipose tissue, while not producing ERα agonist effects on mouse MG and uterus, and human MCF-7 cell xenografts.

The non-selective tissue agonist action of E2 was also demonstrated at a genomic level using microarrays as E2 regulated 1,179 genes in adipose tissue, 2,239 genes in the uterus and 479 genes in the MG. In contrast, RG and RP regulated genes in a tissue-specific manner. RG and RP regulated a similar number of genes in adipose tissue as E2, but much fewer genes in MG and uterus. The overlap of regulated genes between E2 and RG or RP was also much greater in adipose tissue compared to MG and uterus. 24% of genes regulated by RG and RP in adipose tissue overlapped with those regulated by E2, whereas there was only 0–2% overlap in the MG and a 4–11% overlap in the uterus. It is likely that different genes regulated by RG and RP compared to E2 account for their tissue selective agonist actions. These findings demonstrate that RG and RP mimic the effects of E2 on gene expression more strongly in adipose tissue than the MG and uterus.

The mechanisms whereby RG and RP exert tissue-specific effects are unknown. One possibility is selective tissue uptake of the compounds. For example, an active ER-binding compound in these extracts is diffused or transported to the fat but not to the MG or uterus. Another possibility is that RG and RP might activate ERα in the adipose tissue and not in the mammary gland or uterus where it is known to promote proliferation. This could occur if RG and RP binding to ERα causes a recruitment of tissue-specific coregulators or transcription factors required for regulating gene transcription. For example, the coactivator NCOA1 is highly expressed in endometrial cancer cells, but is present in low levels in breast cancer cells. The higher expression of NCOA1 allows tamoxifen to act as an ERα agonist in endometrial cancer, but not in breast cancer cells [47]. Northern blot analysis showed distinct tissue-specific expression patterns of coregulatory genes [48]. There is also tissue-specific expression of coregulator proteins in reproductive organs [49]. These findings suggest a major determinant of the tissue-specific response to estrogens is the expression of different coregulator proteins in tissues. Another possibility is that some other chemicals in the extracts block ERα action in MG and uterus, but not in the adipose tissue. These issues will require further study.

Our microarray analysis of adipose tissue identified several genes commonly regulated by E2, RG and RP that might be involved in mediating the reversal of weight gain and fat accumulation. GPX3 might be an important mediator of estrogen effects in relation to fat accumulation because GPX3 levels are lower in obesity and higher after weight loss [33]. One family of genes regulated by E2, RG and RP that also may be important in mediating the anti-obesity effects encode for the major urinary proteins (MUPs), which regulate systemic glucose and lipid metabolism through paracrine/autocrine regulation of liver metabolic activity [50].

Since RG and RP are crude extracts it is possible that their effects on weight gain, fat accumulation and gene regulation are produced by compounds that affect other cellular pathways, rather than ER signaling. A lack of an effect on ERα could explain why RG and RP do not cause proliferation in the mammary gland and uterus. It is conceivable that some of the effects of RG and RP were mediated through ERβ because ERβ agonists do not stimulate the mammary gland and uterus and the ERβ agonist, MF101, produced a modest, but statistically significant reduction in both weight and body mass index in postmenopausal women, compared to placebo after 12 weeks of treatment [25]. Furthermore, we observed that RG and RP can activate ERβ in transfection assays (data not shown). However, it is unlikely that effects of RG and RP on weight and fat accumulation are mediated through ERβ. First, mouse adipose tissue expressed mainly ERα. Second, the *in vivo* gene expression profiling analysis shows that RG and RP and E2 regulate approximately 1000 genes in fat tissue, whereas in the mammary gland tissue RG and RP regulated 0 and 22 genes, respectively even though it expresses ERβ [51]. The lack of genes regulated in the MG indicates that RG and RP do not regulate ERα or ERβ in the mammary gland. Third, RG and RP regulated 229 and 664 genes, respectively in the mouse uterus which expresses very little ERβ [52] indicating that these genes were likely regulated by ERα. However, it is possible that ERβ-selective drugs may exert beneficial effect on obesity and metabolism indirectly through regulation of other tissues and not through direct effects on adipocytes. It is also possible that the effects of RG and RP are mediated by a pathway that does not involve ERs, but this seems unlikely because no genes were regulated in U2OS cells that did not express ERα, whereas many genes were regulated by RG and RP when ERα was present. Although crude plant extracts were used in these studies it is clear that RG and RP show promising tissue selective agonist activity, and are worthy of further investigation to characterize the pure active compounds and verify that the effects of RG and RP on adipose tissue are mediated by ERα. Our findings demonstrate that the plant extracts have estrogenic activity and reverse weight gain and fat accumulation without stimulating the proliferation of the mouse mammary gland and uterus. RG, RP and their active compounds might constitute a new class of tissue selective estrogen agonists to prevent previous indications for MHT requiring long-term use, such as osteoporosis, as well as new indications, such as obesity, type 2 diabetes, and metabolic syndrome.

## Supporting Information

Table S1 Genes regulated by RG and RP in U2OS-ERα cells maintained in the absence (−) and presence (+) of doxycycline (dox) as described in the legend of Table 1.
(XLS)

Table S2 RG and RP do not alter feeding intake. Food consumption was measured per cage during the 7 weeks of treatment. Normalized daily food intake in mg food/g body weight/day is given for each week of treatment.
(DOC)

Table S3 Genes regulated by E2, RG and RP in mouse gonadal fat (Fat), mammary gland (MG) and uterus (Ut) as described in the legend of Table 2.
(XLS)
