# Supplementary material for: Estrogenic Plant Extracts Reverse Weight Gain and Fat Accumulation without Causing Mammary Gland or Uterine Proliferation
Source: PLoS One. 2011 Dec 7;6(12):e28333. doi: 10.1371/journal.pone.0028333 (PMC3233562; doi:10.1371/journal.pone.0028333)
Supplement: Table S2 — RG and RP do not alter feeding intake. Food consumption was measured per cage during the 7 weeks of treatment. Normalized daily food intake in mg food/g body weight/day is given for each week of treatment. (DOC) [file pone.0028333.s002.doc]

| Treatment | Week 1 | Week 2 | Week 3 | Week 4 | Week 5 | Week 6 | Week 7 |
| --- | --- | --- | --- | --- | --- | --- | --- |
| Control | 0.046682 | 0.078601 | 0.055775 | 0.062321 | 0.057166 | 0.062909 | 0.046221 |
| E2 | 0.019421 | 0.055229 | 0.064265 | 0.077593 | 0.076701 | 0.08557 | 0.078675 |
| RG | 0.049264 | 0.048515 | 0.054955 | 0.05579 | 0.052511 | 0.058899 | 0.061122 |
| RP | 0.032464 | 0.051808 | 0.053568 | 0.050946 | 0.051955 | 0.052956 | 0.058313 |

**Table S2**
